# Barbaloin Protects Pentylenetetrazol-Induced Cognitive Deficits in Rodents via Modulation of Neurotransmitters and Inhibition of Oxidative-Free-Radicals-Led Inflammation

**DOI:** 10.3390/ph17060699

**Published:** 2024-05-28

**Authors:** Ahmad Essam Altyar, Muhammad Afzal, Nehmat Ghaboura, Khalid Saad Alharbi, Sattam Khulaif Alenezi, Nadeem Sayyed, Imran Kazmi

**Affiliations:** 1 Department of Pharmacy Practice, Faculty of Pharmacy, King Abdulaziz University, P.O. Box 80260, Jeddah 21589, Saudi Arabia; 2 Pharmacy Program, Batterjee Medical College, P.O. Box 6231, Jeedah 21442, Saudi Arabia; 3 Department of Pharmaceutical Sciences, Pharmacy Program, Batterjee Medical College, P.O. Box 6231, Jeedah 21442, Saudi Arabia; 4 Department of Pharmacy Practice, Pharmacy Program, Batterjee Medical College, P.O. Box 6231, Jeedah 21442, Saudi Arabia; pharmacy8.jed@bmc.edu.sa; 5 Department of Pharmacology and Toxicology, College of Pharmacy, Qassim University, Buraydah, Al Qassim 51452, Saudi Arabia; khalid.alharbi9@qu.edu.sa (K.S.A.); sk.alenezi@qu.edu.sa (S.K.A.); 6 Glocal School of Pharmacy, Glocal University, Mirzapur-Pole, Saharanpur 247121, India; snadeem.pharma@gmail.com; 7 Department of Biochemistry, Faculty of Science, King Abdulaziz University, P.O. Box. 80200, Jeddah 21589, Saudi Arabia; ikazmi@kau.edu.sa

**Keywords:** barbaloin, pentylenetetrazol kindling, cytokines, neuroprotective, oxidative stress

## Abstract

Background: Epilepsy is defined by an excessive level of activity in the neurons and coordinated bursts of electrical activity, resulting in the occurrence of seizure episodes. The precise cause of epileptogenesis remains uncertain; nevertheless, the etiology of epilepsy may involve neuroinflammation, oxidative stress, and malfunction of the neurotransmitter system. Objective: The goal of this investigation was to assess barbaloin’s protective properties with respect to pentylenetetrazol (PTZ)-)-induced cognitive deficits in rats via antioxidative, anti-inflammatory, and neurotransmitter-modulating effects. Methods: *Wistar* rats were subjected to PTZ [40 mg/kg (i.p.)], which induced cognitive decline. Behavior assessment using a kindling score, open-field test (OFT), novel object recognition test (NORT), and assays for *superoxide dismutase (SOD), reduced glutathione (GSH), catalase (CAT), malondialdehyde (MDA), acetylcholinesterase (AChE), caspase-3, nitric oxide (NO), interleukins-1β (IL-1β)*, *tumor necrosis factor-α (TNF-α), IL-6, nuclear factor kappa-B (NF-κB), Bcl-2* and *Bax*, and neurotransmitter levels [GABA, DA, NE, and serotonin (5-HT)] were performed. Results: The treatment of rats with barbaloin resulted in behavior improvement and significant changes in the levels of *GSH, SOD, CAT, MDA, AChE, NO*, *IL-6, IL-1β, TNF-α, NF-κB, caspase-3, Bcl-2,* and *Bax* compared to the PTZ control group. Barbaloin treatment resulted in notable changes in neurotransmitter levels *(GABA, NE, 5-HT, DA*) compared to the PTZ group. Conclusions: The ongoing study has gathered evidence indicating that the injection of barbaloin has resulted in significant improvements in cognitive performance in rats. This is achieved by inhibiting oxidative stress, enhancing the activity of natural antioxidant enzymes, reducing cytokine levels, and increasing the levels of neurotransmitters in the brain. These results were detected in comparison to a PTZ control and can be attributed to the potent anti-inflammatory and antioxidant capabilities of barbaloin, which could be linked to its neuroprotective properties. Barbaloin may potentially increase cognitive decline and boost neuronal survival by altering the expression of *Bax, caspase-3, Bcl-2.*

## 1. Introduction

Epilepsy is a widespread neurological condition that impacts around 1% of the global population, with a significantly greater occurrence in poorer nations [1]. Epilepsy is defined by an excessive level of activity in the neurons and coordinated bursts of electrical activity, resulting in the occurrence of seizure episodes [2]. Multiple sources of evidence have shown that inflammatory markers enhance the excitability of neurons, increase the blood–brain barrier’s (BBR) permeability, activate glial cells, and induce neuronal death. Evidence demonstrates that the secretion of cytokines and chemokines by activated glial cells, specifically astrocytes and microglia, is highly significant in the development and advancement of neurological illnesses such as epilepsy [3,4,5]. The precise cause of epileptogenesis remains uncertain; nevertheless, the etiology of epilepsy may involve neuroinflammation, oxidative stress, and malfunction of the neurotransmitter system [6].

The transcription factor *Nrf2* controls antioxidant function by inducing the expression of many antioxidant enzymes, like reduced glutathione (GSH) and superoxide dismutase (SOD)-related enzymes. Consequently, the *Nrf2* molecule was specifically focused on during the creation of anti-epileptic medications [7]. Furthermore, the presence of neuroinflammation is indicated by the activation of neuroglial cells and the release of cytokines. This has been proposed to intensify epileptic convulsions. Reported cytokines such as *nuclear factor kappa B (NF-κB), GFAP, tumor necrosis factor-α (TNF-α),* and *IL-6* have been shown to produce neuronal hyperexcitability, leading to the development of seizures [8,9].

Pentylenetetrazol (PTZ)-kindling is a long-term model of epilepsy, a condition marked by a progressive escalation of seizures. PTZ-induced kindling induces alterations occurring at the cellular and molecular level, which contribute to neural plasticity [10,11,12]. The induction of the kindling model is achieved with the repeated administration of PTZ as an antagonist of γ-aminobutyric acid type A (GABA-A) at sub-convulsive dosages [13,14]. Studies have demonstrated that the injection of PTZ leads to the death of neurons and the activation of glial cells in particular areas of the hippocampus [15]. Furthermore, the researchers employed PTZ kindling, a well-established paradigm commonly utilized to investigate the processes behind epileptogenesis, as well as the cognitive impairments resulting from seizures [16,17]. With repeated PTZ administration, each injection triggers a seizure, and the severity of the seizures gradually increases [18]. Preclinical and clinical research provide compelling data indicating that epilepsy may lead to neuroinflammation [19]. Excessive inflammatory processes are linked to impaired neuronal function, and proinflammatory cytokines contribute to the development of seizures by triggering the infiltration of white blood cells, disrupting the blood–brain barrier and boosting the oxidation of lipids [20,21].

Herbal medications provide promising and valuable reservoirs of medicinal compounds [22,23]. Barbaloin is an organic compound with bioactive properties that is derived from the *Aloe vera* L. plant. Barbaloin, like *A. vera*, has several pharmacological impacts such as anti-inflammatory, antimicrobial, and antioxidant effects [24,25,26]. Prior studies have confirmed that barbaloin triggers the overexpression of *IL-6, tumor necrosis factor-α, and IL-1β* by activating *NF-κB* [26,27,28,29]. Moreover, the impact was notably reduced when the *PI3K/AKT* signaling was obstructed. The previous results provide empirical evidence that substantiates the notion that barbaloin effectively reduces the generation of reactive oxygen species (ROS) in cells by impeding the phosphorylation process of *PI3K* and *AKT.* Consequently, this obstacle inhibits the stimulation of NF-κB [27,28]. The findings suggest that barbaloin possesses antidiabetic, antioxidant, neuroinflammatory, cytokine-inhibitory, and *acetylcholinesterase* (*AChE*)-inhibiting properties. These properties may contribute to its ability to protect against cognitive decline caused by STZ. Barbaloin may have potential clinical applications in the treatment of neurological and cognitive deficits in individuals with diabetes [28]. Barbaloin’s ability to improve cognitive function stems from its ability to inhibit oxidative stress and enhance the activity of endogenous antioxidant enzymes within the brain [30]. By minimizing the damage caused by oxidative stress, barbaloin can preserve the integrity of the brain’s structure and function, leading to improved cognitive performance.

There has been no research conducted on the impact of barbaloin in epilepsy animal models. The objective of the current study was to investigate the impact of barbaloin on seizure tendencies and cognitive decline in a PTZ-induced kindling paradigm. In addition, the concentration of inflammation, as well *as caspase-3, Bcl-2-associated X protein (Bax), B-cell lymphoma 2 (Bcl-2) marker*, and neurotransmitter content in the brain, were assessed in rats treated with barbaloin. The observed results were compared to a PTZ control and can be attributed to the potent anti-neuroinflammatory and oxidative stress decline capabilities of barbaloin, which may enhance its ability to protect against neuronal damage.

## 2. Results

### 2.1. Kindling Score

**Figure 1:**
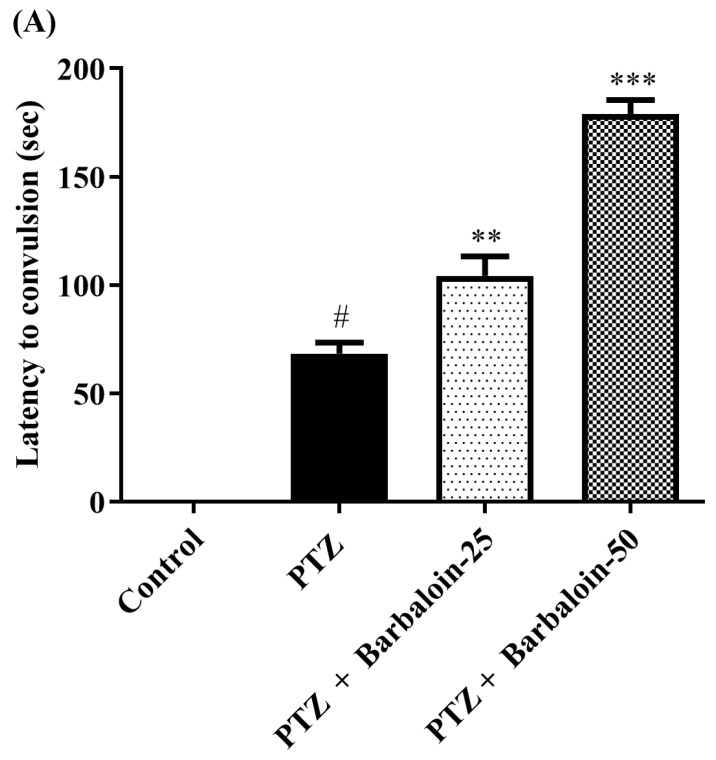

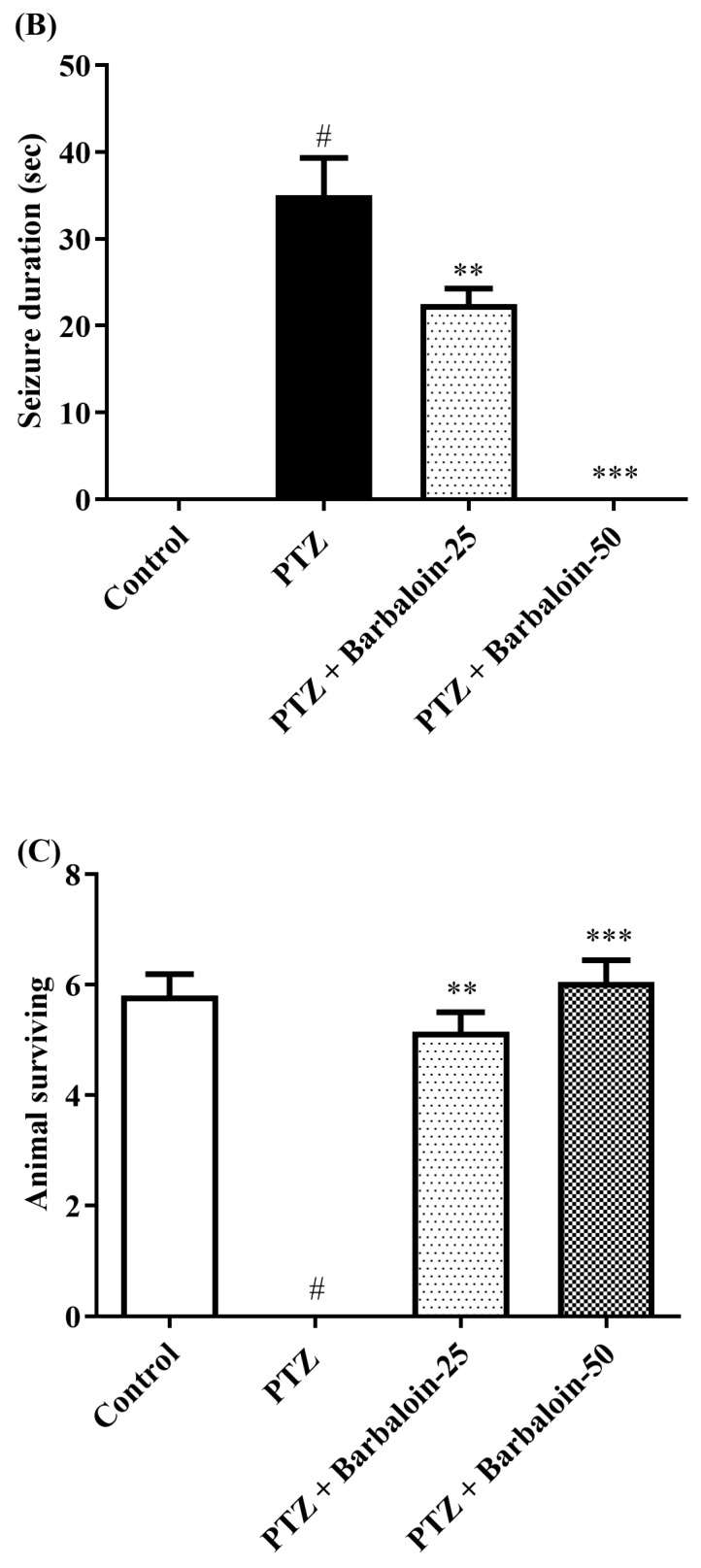
(**A**–**C**) Outcome of barbaloin on kindling score. Mean ± S.E.M (*n* = 6). One-way ANOVA followed by Tukey’s post hoc test (*n* = 6). # *p* < 0.001 vs. control, ** *p* < 0.001, *** *p* < 0.0001 vs. PTZ.

The administration of PTZ induced generalized tonic–clonic seizures (GTCS), which were assessed using the Racine scale. According to the data shown in Figure 1A–C, the administration of barbaloin for a period of 4 weeks significantly reduced the duration of seizures [F (3, 20) = 55.54, *p* < 0.0001]. The treated groups had a significantly prolonged latent time before the onset of seizures compared to the PTZ control rats [F (3, 20) = 148.7, *p* < 0.0001]. Furthermore, the mortality rate was significantly decreased in the treated groups compared to the PTZ control group [F (3, 20) = 76.09, *p* < 0.0001]. The findings indicate that barbaloin has anticonvulsant properties against PTZ-induced seizures.

### 2.2. Open Field Test (OFT)

**Figure 2:**
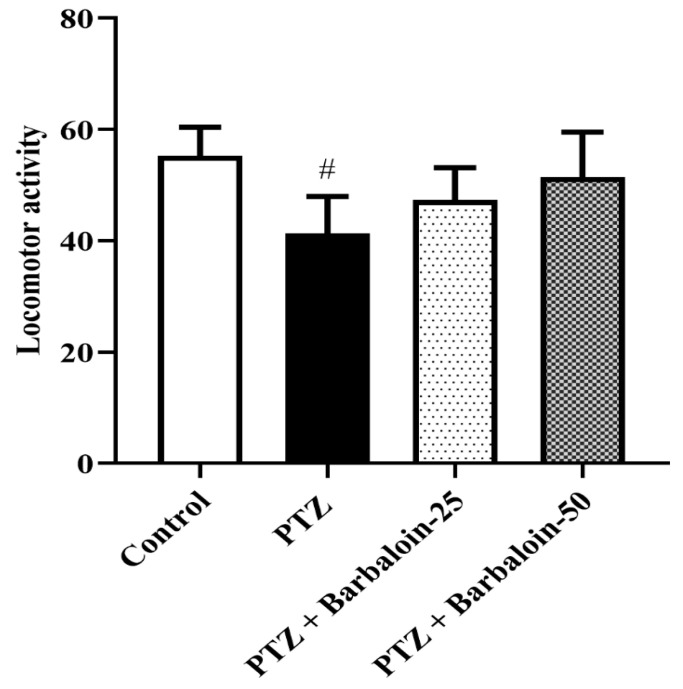
The effect of barbaloin on OFT. Mean ± S.E.M. One-way ANOVA followed by Tukey’s post hoc test (*n* = 6). # *p* < 0.001 vs. control.

PTZ control rats had no significant effect on exploratory and locomotor in comparison to rats in normal control (*p* < 0.0001). Treatment with barbaloin [F (3, 20) = 0.8348, *p* = 0.4905] also had no significant effect compared to PTZ control rats (Figure 2).

### 2.3. Novel Object Recognition Test (NORT)

**Figure 3:**
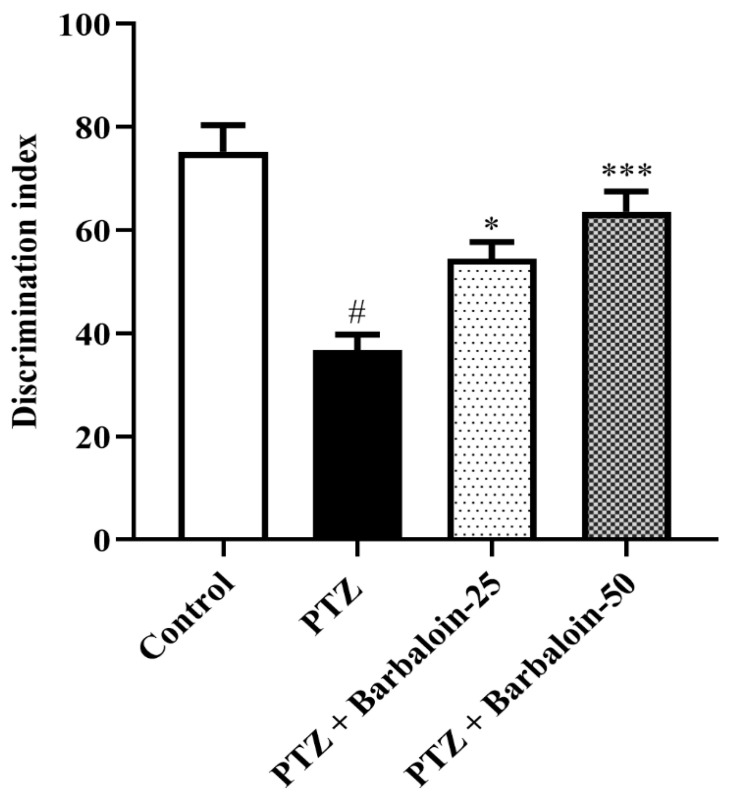
The effect of barbaloin on NORT. Mean ± S.E.M. One-way ANOVA followed by Tukey’s post hoc test (*n* = 6). # *p* < 0.001 vs. control, * *p* < 0.05, *** *p* < 0.0001 vs. PTZ.

PTZ control rats displayed a significantly decreased discrimination index in comparison to normal control rats (*p* < 0.0001). Treatment with barbaloin enhanced the discrimination index [F (3, 20) = 16.87, *p* < 0.0001] compared to PTZ control rats (Figure 3).

### 2.4. Malondialdehyde (MDA), Nitric Oxide (NO), AChE Estimation

**Figure 4:**
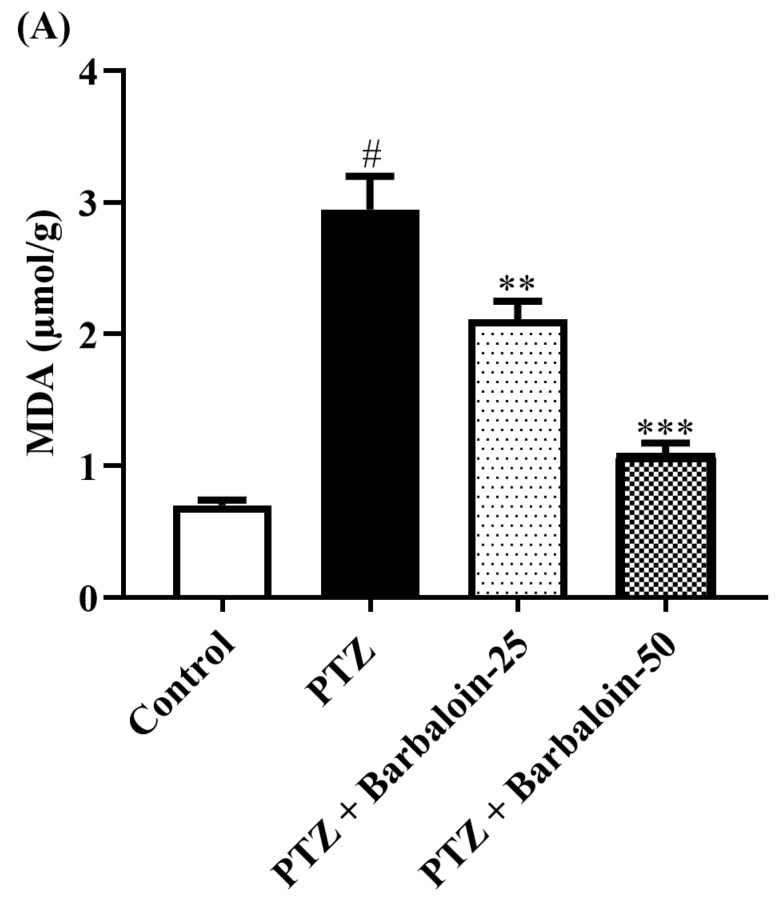

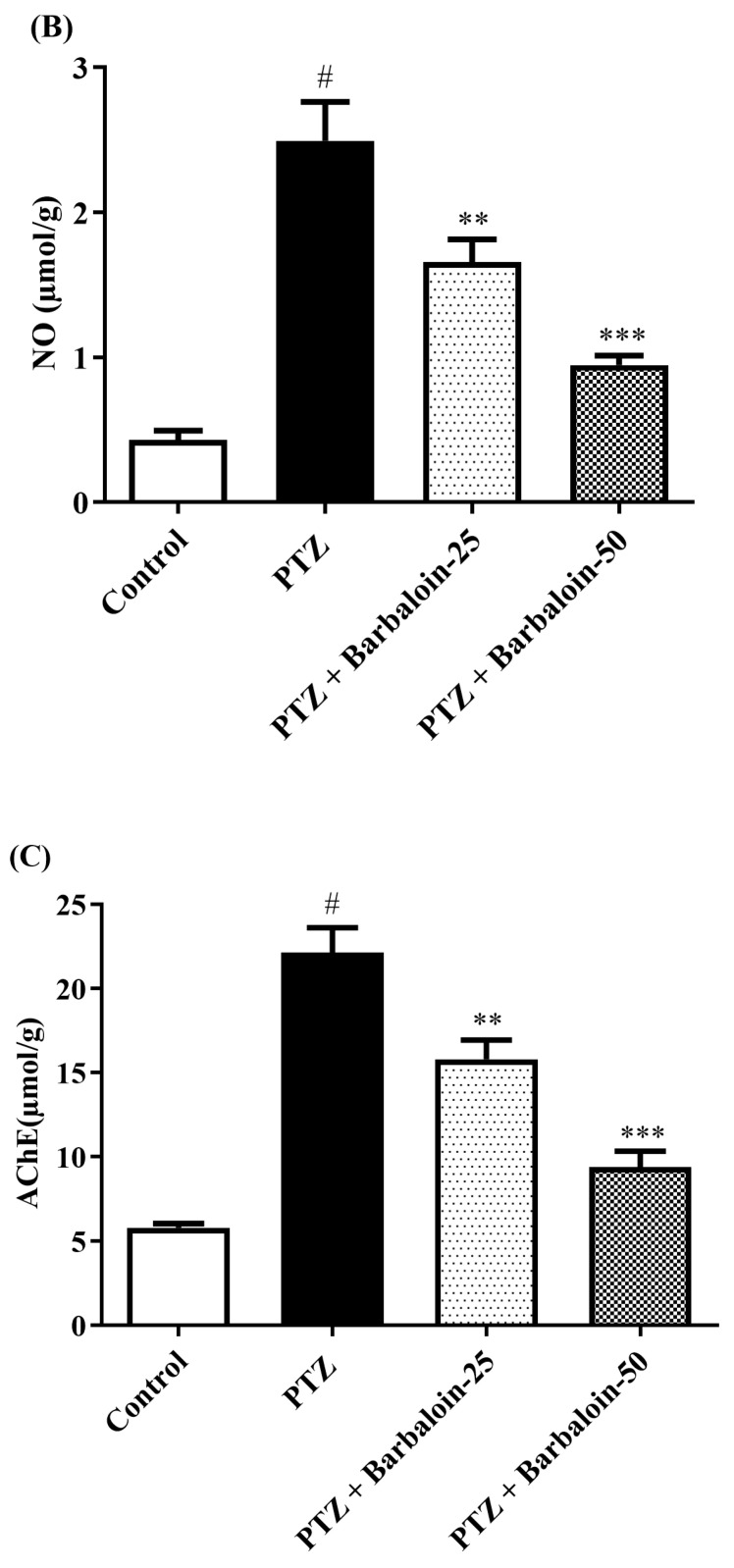
(**A**–**C**) The effect of barbaloin on *MDA, NO,* and *AChE* levels. Mean ± S.E.M. One-way ANOVA followed by Tukey’s post hoc test (*n* = 6). # *p* < 0.001 vs. control, ** *p* < 0.001, *** *p* < 0.0001 vs. PTZ.

PTZ control rats significantly enhanced brain *MDA, NO,* and *AChE* concentration in comparison to rats in normal control (*p* < 0.0001). Treatment with barbaloin lowered the *MDA* [F (3, 20) = 45.40, *p* < 0.0001], NO [F (3, 20) = 30.54, *p* < 0.0001], and *AChE* [F (3, 20) = 46.54, *p* < 0.0001] levels, respectively, compared to PTZ control rats (Figure 4A–C).

### 2.5. SOD, GSH, Catalase (CAT) Estimation

**Figure 5:**
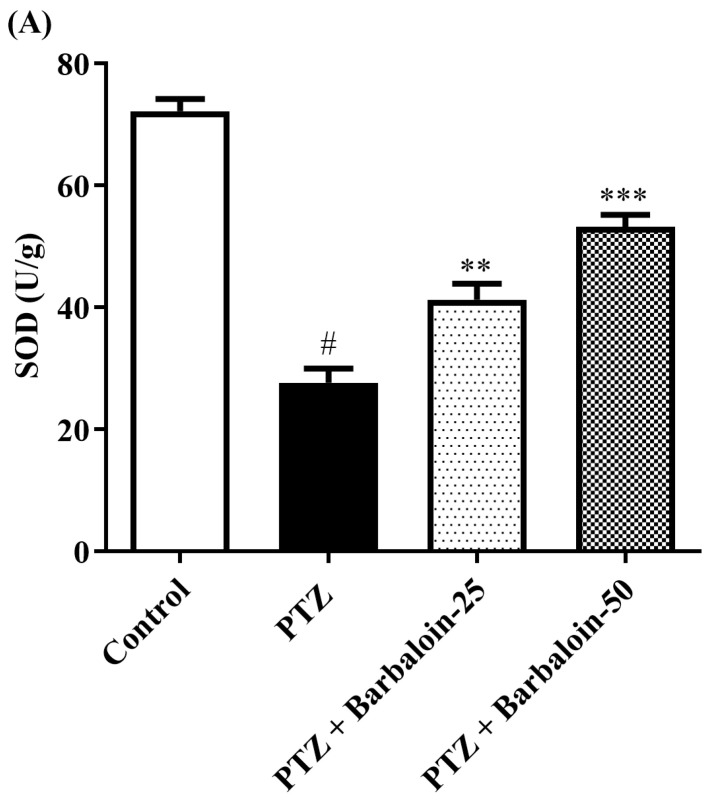

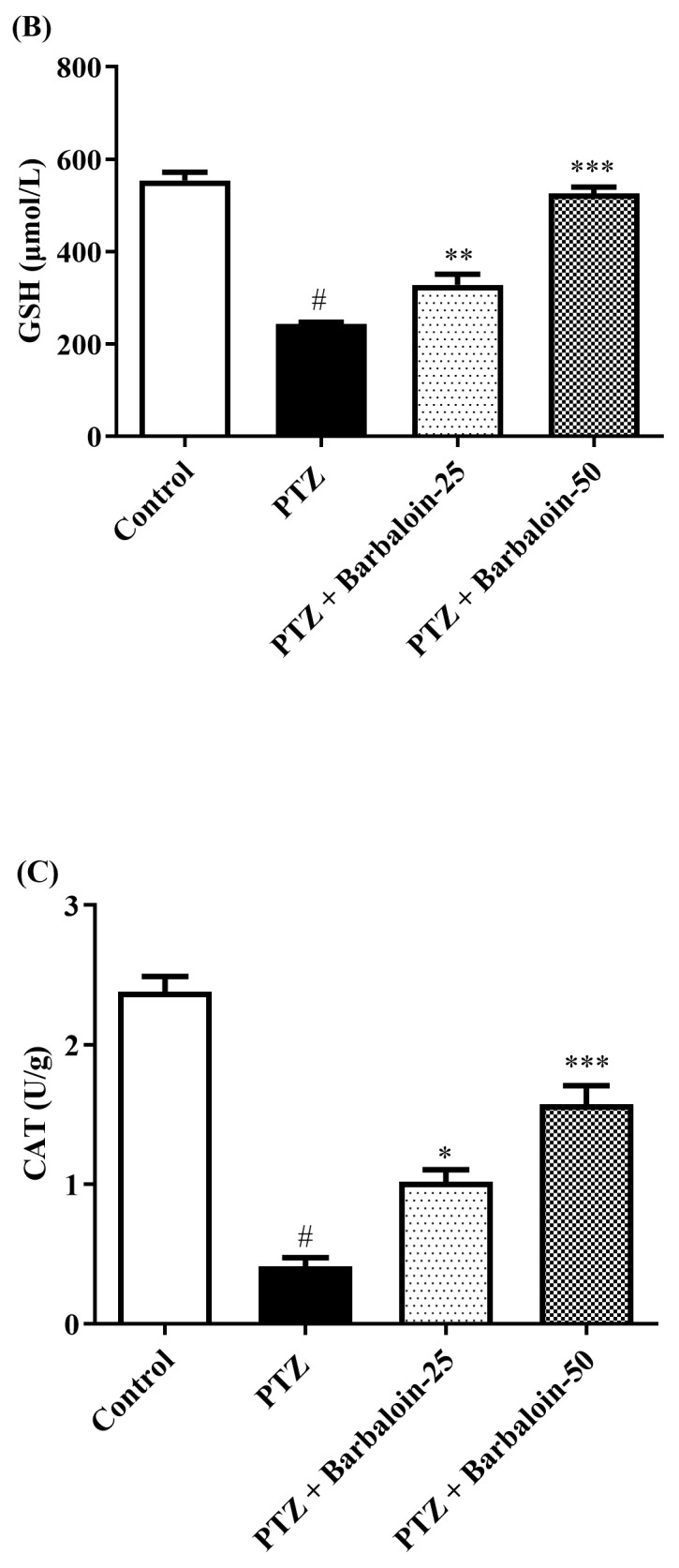
(**A**–**C**) The effect of barbaloin on *SOD, GSH, and CAT levels*. Mean ± S.E.M. One-way ANOVA followed by Tukey’s post hoc test (*n* = 6). # *p* < 0.001 vs. control, * *p* < 0.05, ** *p* < 0.001, *** *p* < 0.0001 vs. PTZ.

PTZ control rats significantly decreased brain *SOD, GSH,* and *CAT* concentration in comparison to rats in normal control (*p* < 0.0001). Treatment with barbaloin enhanced brain *SOD* [F (3, 20) = 70.84, *p* < 0.0001], *GSH* [F (3, 20) = 86.95, *p* < 0.0001], and *CAT* [F (3, 20) = 68.84, *p* < 0.0001] levels, respectively, compared to PTZ control rats (Figure 5A–C).

### 2.6. Brain GABA, Dopamine (DA), Norepinephrine (NE), and 5-Hydroxytryptamine (5-HT) Contents

**Figure 6:**
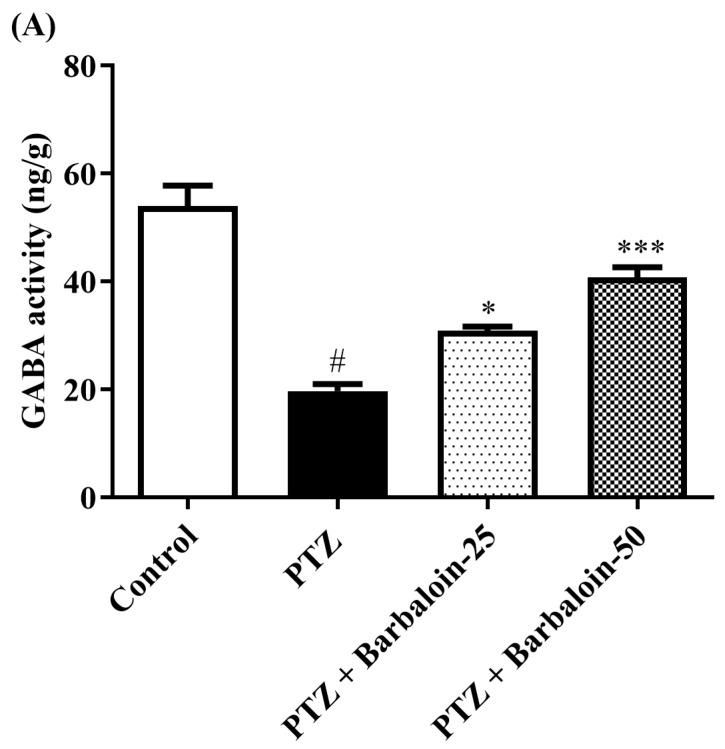

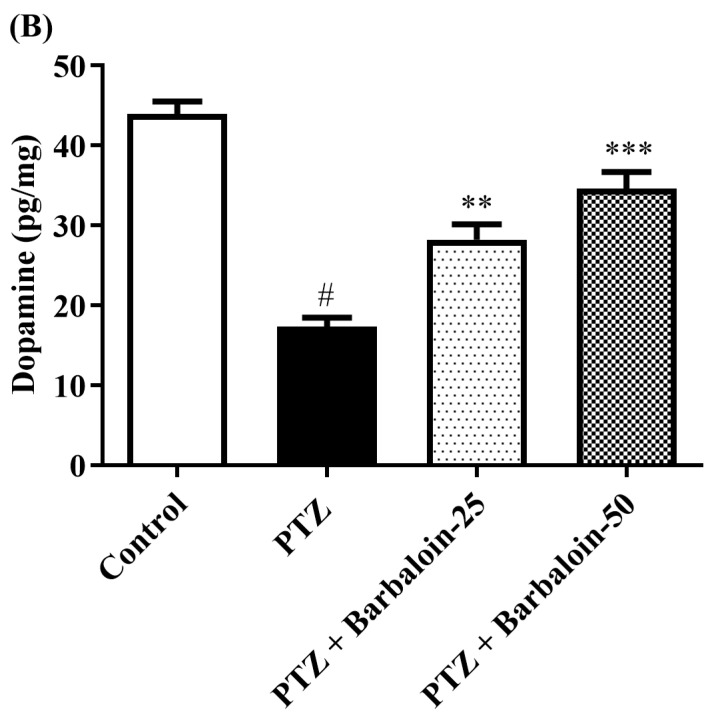

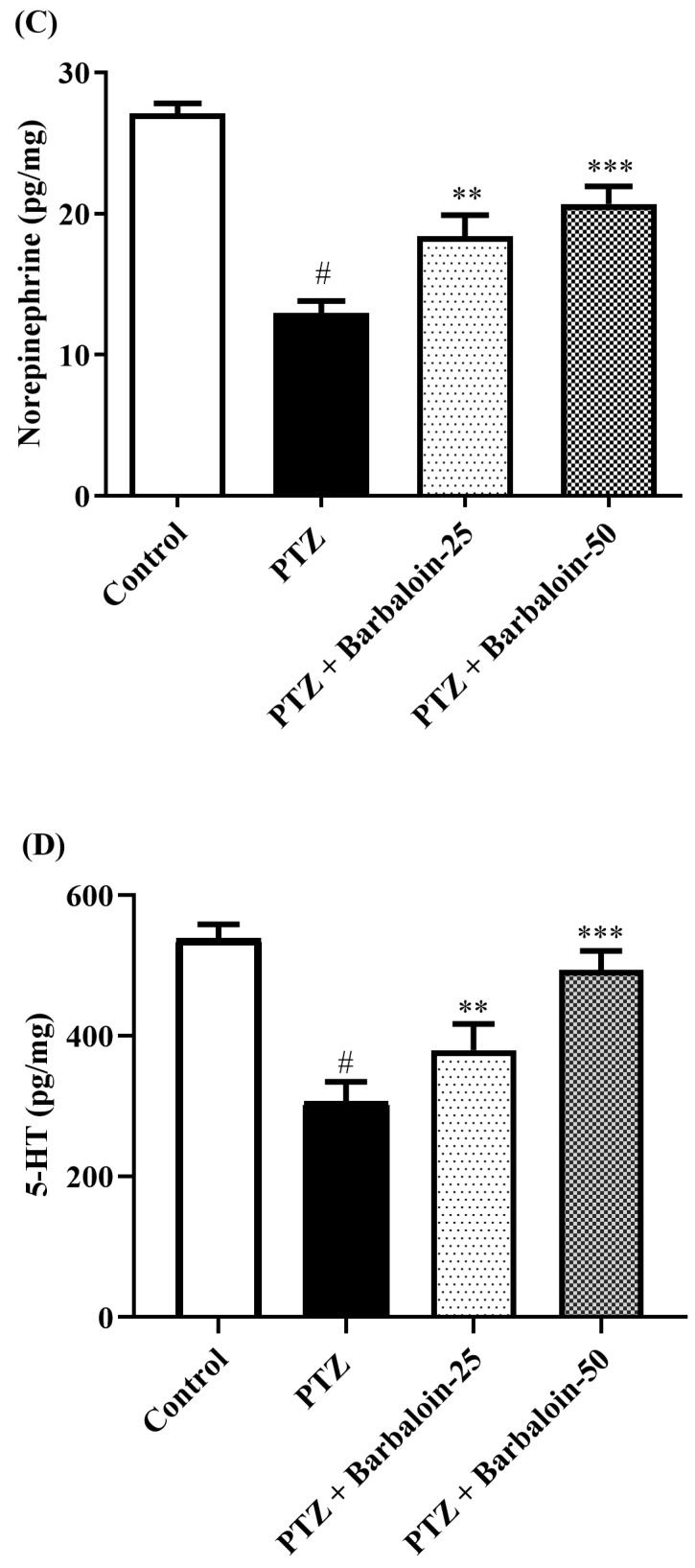
(**A**–**D**) The effect of barbaloin on GABA, DA, NE, and 5-HT levels. Mean ± S.E.M. One-way ANOVA followed by Tukey’s post hoc test (*n* = 6). # *p* < 0.001 vs. control, * *p* < 0.05, ** *p* < 0.001, *** *p* < 0.0001 vs. PTZ.

Under comparable circumstances, PTZ control rats exhibited a significant decrease in GABA, DA, NE, and 5-HT levels in the brain compared to the normal control (*p* < 0.0001). Treatment with barbaloin enhanced the GABA [F (3, 20) = 41.59, *p* < 0.0001], DA [F (3, 20) = 42.39, *p* < 0.0001], NE [F (3, 20) = 26.74, *p* < 0.0001], and 5-HT [F (3, 20) = 79.84, *p* < 0.0001] contents in comparison to PTZ control rats (Figure 6A–D).

### 2.7. Estimation of Cytokines

**Figure 7:**
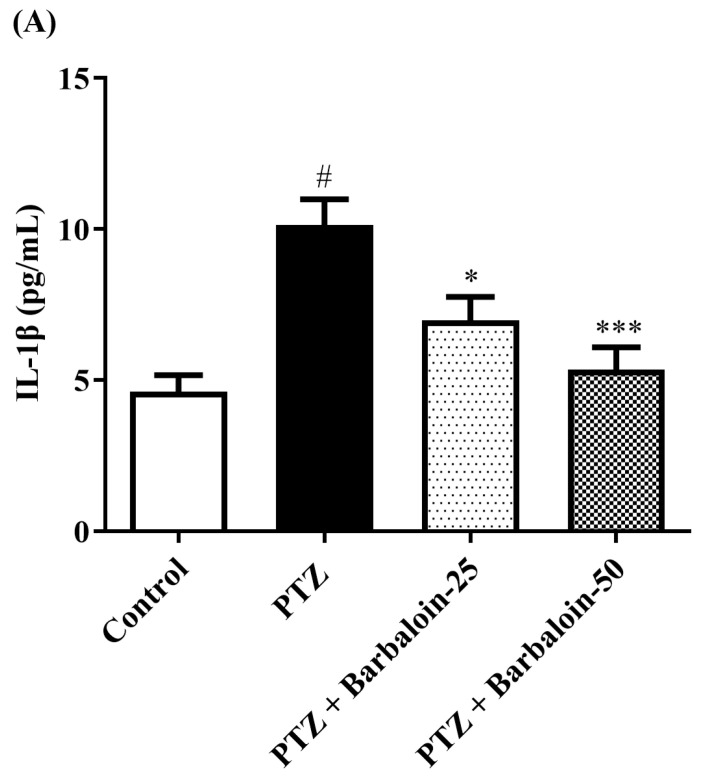

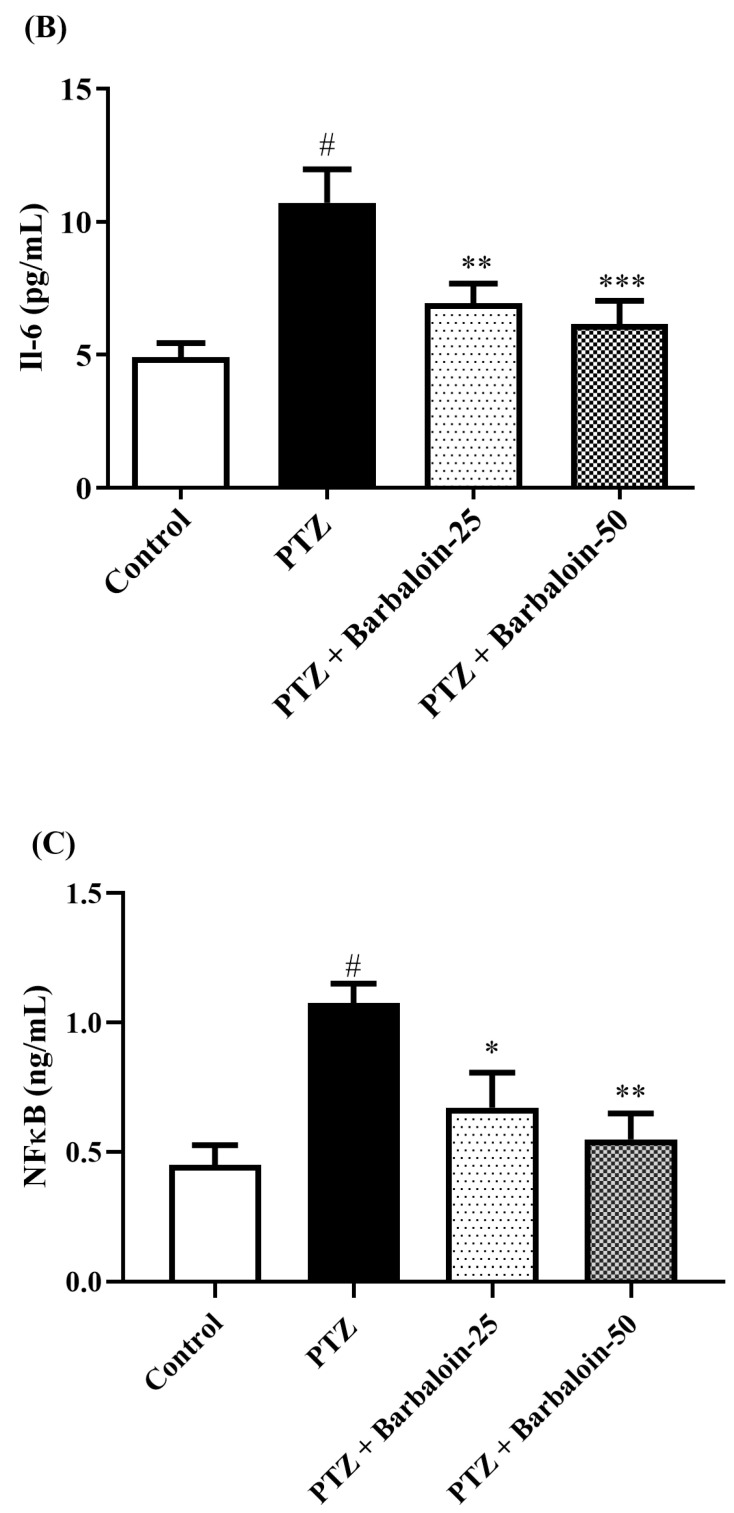

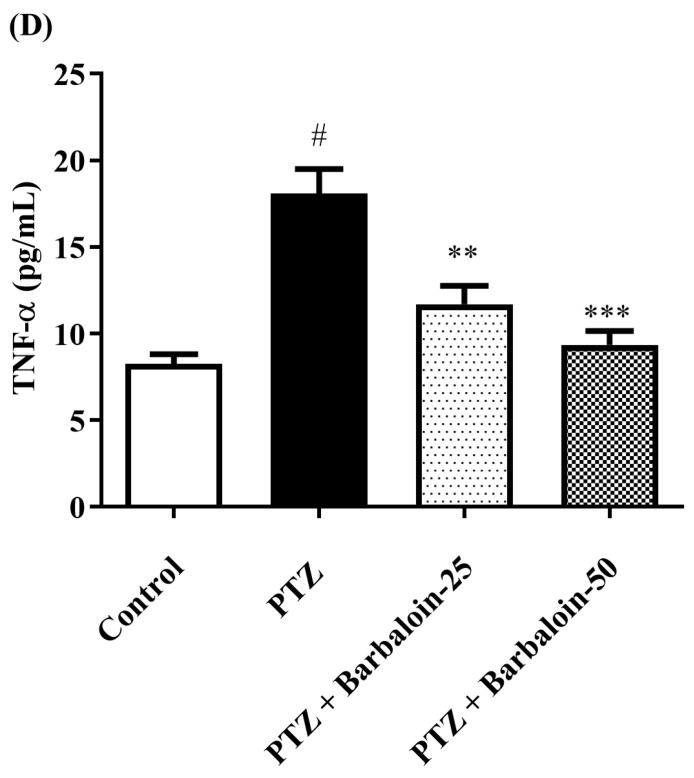
(**A**–**D**) The effect of barbaloin on *IL-1β, IL-6, NF-κB, and TNF-α* levels. Mean ± S.E.M. One-way ANOVA followed by Tukey’s post hoc test (*n* = 6). # *p* < 0.001 vs. control, * *p* < 0.05, ** *p* < 0.001, *** *p* < 0.0001 vs. PTZ.

Cytokines IL-6, IL-1β, NF-κB, and TNF-α were significantly increased in the PTZ control in comparison to rats in normal control (*p* < 0.0001). Administration of barbaloin seemingly declines the *IL-1β* [F (3, 20) = 10.99, *p* = 0.0002], *IL-6* [F (3, 20) = 7.912, *p* = 0.0011], *NF-κB* [F (3, 20) = 7.573, *p* = 0.0014], and *TNF-α* [F (3, 20) = 18.38, *p* < 0.0001] levels in comparison to PTZ control. Figure 7A–D display the outcomes acquired from the *IL-1β, IL-6, NF-κB, and TNF-α* examination.

### 2.8. Caspase-3 Bcl-2 and Bax Contents

**Figure 8:**
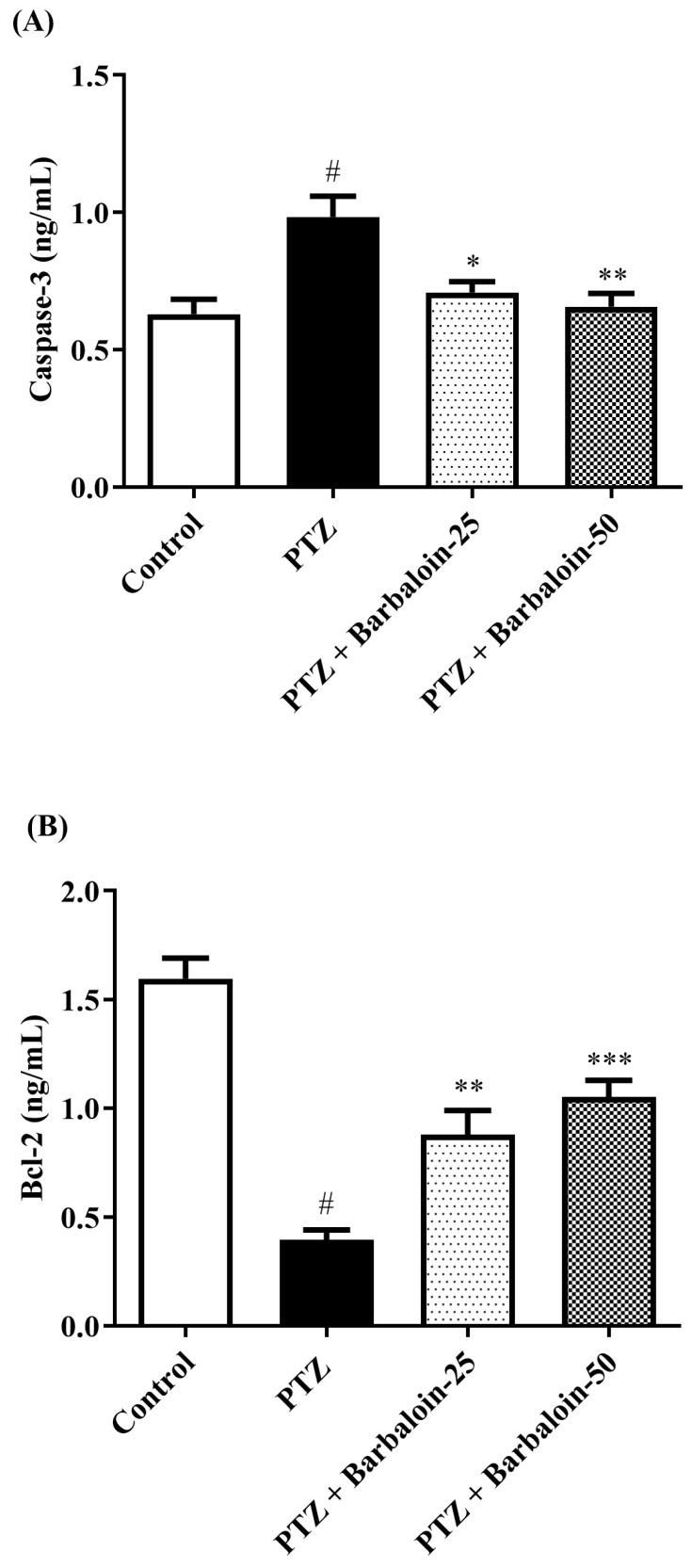

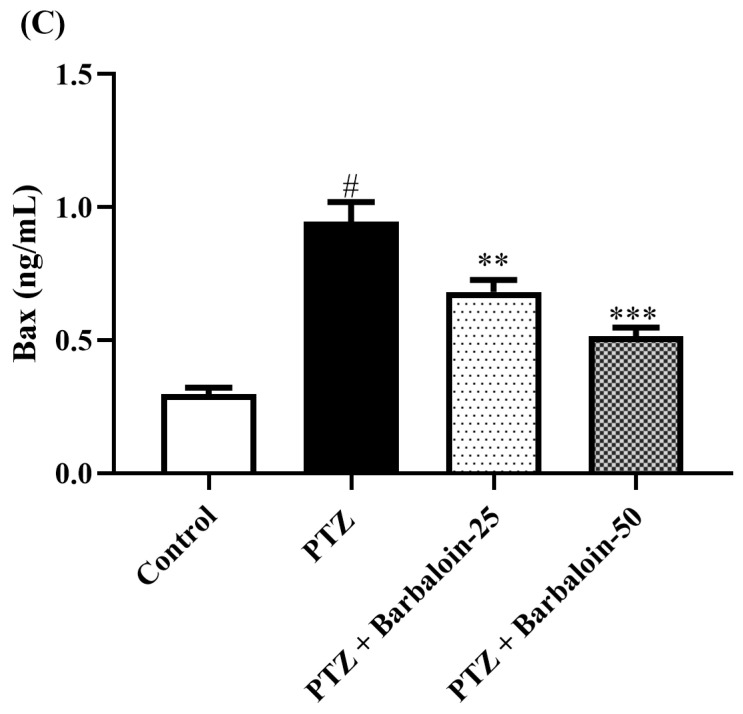
(**A**–**C**) The effect of barbaloin on *caspase-3, Bcl-2,* and *Bax* levels. Mean ± S.E.M. One-way ANOVA followed by Tukey’s post hoc test (*n* = 6). # *p* < 0.001 vs. control, * *p* < 0.05, ** *p* < 0.001, *** *p* < 0.0001 vs. PTZ.

PTZ control rats considerably upregulated brain *caspase-3* and *Bax* and downregulated *Bcl-2* expression in comparison to rats in normal control (*p* < 0.0001). Barbaloin-treatment downregulated *Bax* [F (3, 20) = 32.04, *p* < 0.0001] and *caspase-3* [F (3, 20) = 8.379, *p* = 0.0008] and upregulated the Bcl-2 [F (3, 20) = 33.65, *p* < 0.0001] level, respectively, in comparison to PTZ control rats (Figure 8A–C).

## 3. Discussion

This study aimed to assess the potential neuroprotective impact of barbaloin in rats with PTZ-induced kindling, which leads to cognitive decline. The ingestion of PTZ was observed to impede these benefits. This was achieved by examining changes in kindling score, open field test, NORT, oxidative stress levels, cytokine levels, and the expression of proteins *caspase-3, Bcl-2,* and *Bax*. The results indicate that the administration of barbaloin maintained the kindling score, as well as in the levels of *NO, GSH, MDA, CAT, AChE, SOD, TNF-α, Bcl-2, IL-6, Bax, IL-1β, NF-κB,* and *caspase-3*. Furthermore, the barbaloin also led to significant enhancements in the level of neurotransmitters such as 5-HT, DA, NE, and GABA.

The experiment demonstrated that the PTZ-treated animals interacted more similarly with both familiar and novel objects and were unable to recall the familiar object, as seen by the drop in the discrimination index in the NORT, whereas treatment with barbaloin enhanced the discrimination index. According to studies, the hippocampus is important for memory related to object recognition. Anterograde memory will alter modestly and consistently if this structure is compromised [31]. In OFT, the PTZ-induced and barbaloin treatment group had no significant effect on locomotor and exploratory activity. These findings suggest that the PTZ and barbaloin treatment may not have a direct impact on locomotor and exploratory activity.

Oxidative stress is well recognized as one of the primary and essential factors that lead to recurrent seizure occurrence. Excitotoxicity results in the release of free radicals and ROS. Consequently, lipids, proteins, and DNA are oxidized, leading to changes in membrane permeability, protein function, and gene expression [32]. These alterations can make neurons more susceptible to degeneration or mortality [32,33]. According to this study, PTZ kindling leads to a reduction in GSH and elevation in MDA levels. This might happen either via the direct removal of ROS or by promoting the formation of GSH [34,35]. Moreover, PTZ has the ability to universally elevate the concentration of NO throughout the whole brain [36]. In addition, our findings align with these prior studies. This concept is reinforced by the evidence that some medically prescribed antiepileptic medicines (AEDs) decrease ROS during seizures [37], whilst numerous others enhance oxidative harm [38,39]. Therefore, it may be inferred that the use of antioxidants as a supplementary treatment with AEDs can be advantageous in the control of epilepsy, as previously proven [40]. Our investigation found a reduced concentration of GSH, CAT, and SOD, consistent with previous findings suggesting that PTZ treatment causes oxidative stress [41,42]. The current study suggests that rats treated with PTZ exhibit decreased activities of GSH, SOD, and CAT, together with increased levels of MDA and NO, which are indicative of lipid peroxidation. The barbaloin led to a significant increase in the activities of SOD, GSH, and CAT, while simultaneously reducing the quantity of MDA and nitric oxide. Oxidative stress is caused by free radicals, which can cause cell and tissue damage. Barbaloin’s ability to improve cognitive function stems from its ability to inhibit oxidative stress and enhance the activity of endogenous antioxidant enzymes within the brain [30]. By minimizing the damage caused by oxidative stress, barbaloin can preserve the integrity of the brain’s structure and function, leading to improved behavioral cognitive performance.

The AChE is a crucial enzyme that catalyzes the hydrolysis of ACh, hence terminating cholinergic signaling. Thus, AChE is regarded as a significant therapeutic target, and many AChE-reversible antagonists are now employed in clinical practice to increase memory in patients suffering from neurological conditions and epilepsy [43,44,45]. In this work, AChE concentration was enhanced in the PTZ-injected rats. However, the administration of barbaloin to rats resulted in a significant decrease in AChE levels compared to animals stimulated with PTZ.

Seizure activity is linked to a diverse array of localized metabolic alterations that impact different neurotransmitters, including monoamines and amino acids [46]. The present investigation revealed that PTZ led to a decline in GABA, NE, DA, and 5-HT concentration in the hippocampal area. These findings align with the earlier investigations conducted by Visweswari et al. (2010) [47,48,49]. Monoamines are crucial in the epileptogenesis process, which involves the formation and advancement of epilepsy. An observed decrease in 5-HT level was shown to be linked to a decline in its synaptosomal absorption, the tryptophan hydroxylase activity inhibition, and a decline in tryptophan concentration in the epilepsy model [50,51]. Conversely, the reduction in DA levels observed in individuals with epilepsy can be linked to the heightened activity of monoamine oxidase and the diminished reuptake process [52]. In addition, NE functions as a neuromodulator with anticonvulsant properties. The reduction in NE levels in individuals with epilepsy is attributed to the decrease in the density of α1 receptors in the brain. This drop may possibly be attributed to the decline in the activity of dopamine-β-hydroxylase (DBH), which is the enzyme that limits the rate of norepinephrine production [53]. During the present research, it was shown that treatment with barbaloin effectively reversed the changes in levels of DA, NE, 5-HT, and GABA.

The synthesis of pro-inflammatory indicators, such as *IL-6, TNF-α,* and *IL-1β,* is caused by the activation of *NF-κB* signal transduction through the creation of ROS [54,55]. *IL-1* induces the upregulation of the gene expression of the enzyme *cyclooxygenase-2 (Cox-2),* which is responsible for converting arachidonic acid into prostaglandins. Prostaglandin is a substance that comes before prostacyclin and is involved in inflammatory reactions. It activates astrocytes to generate glutamate, which leads to increased neuroexcitability linked with seizures [56]. The findings demonstrated that the PTZ injection resulted in a neuroinflammatory reaction, as indicated by a substantial rise in the protein concentration of cytokines (*IL-6, TNF-α, NF-κB,* and *IL-1β*) in the cerebral cortex [57]. Furthermore, the injection of PTZ resulted in a considerable rise in *GFAP*, which suggests the activation of astrocytes and subsequent inflammatory and apoptotic reactions in the brain [58]. Our data show that administering barbaloin has a considerable inhibitory effect on the raised levels of *IL-6, TNF-α, IL-1β,* and *NF-κB* in PTZ-induced kindling rats. These substances are known as inflammatory markers.

Preclinical investigations have shown that both transient and oxidative stress and neuronal death may arise from recurrent seizures, which entail molecular-level changes in the hippocampus [59,60]. The collapse of neurons can be attributed to the stimulation of pro-apoptotic proteins, including Bax and caspases 3. The findings from the current study showed that the administration of PTZ led to an enhanced concentration of caspase-3 and Bax and a lower concentration of the Bcl-2. However, these aberrations were reversed and returned to normal levels following treatment with barbaloin. Based on this evidence, barbaloin may inhibit PTZ-induced cognitive decline and changes in rats via modification of cytokines, oxidative stress, and protein expression *Bcl-2, caspases-3,* and *Bax.* PTZ-induced rats exhibited cognitive impairment, as demonstrated by alterations in kindling score, OFT, and NORT. Treatment with barbaloin resulted in significant improvements in cognitive function compared to the PTZ control groups. This suggests that barbaloin has the potential to help with cognitive deficits associated with epilepsy. The administration of PTZ led to changes in oxidative stress markers such as *GSH, MDA, CAT, NO, and SOD*. However, barbaloin treatment substantially restored these markers, indicating its antioxidative properties and its ability to counteract oxidative damage in the brain. In addition, PTZ-induced neuroinflammation was characterized by elevated levels of *NF-κB, IL-1β, TNF-α,* and *IL-6.* But when barbaloin was administered, the levels of these pro-inflammatory cytokines were significantly reduced. This suggests that barbaloin has anti-inflammatory effects that may help mitigate neuroinflammation associated with epilepsy. Moreover, barbaloin treatment caused significant changes in neurotransmitter levels such as *GABA, 5-HT, and DA* compared to PTZ-induced controls. These changes indicate that barbaloin may exert its neuroprotective effects, at least in part, through the modulation of neurotransmitter systems implicated in epilepsy pathogenesis. Lastly, barbaloin altered the expression of apoptotic markers, including *caspase-3, Bcl-2,* and *Bax*, suggesting its potential role in regulating apoptotic pathways and promoting neuronal survival in epileptic conditions. This work employed a minimal number of animals, and in future investigations, histopathology and Western blotting will be necessary to confirm this mechanism.

## 4. Materials and Methods 

### 4.1. Animals

Thirty *Wistar* rats, with a weight range of 275–300 g, were acquired from T. G. Lab, India for this investigation. The participants were housed in a regulated setting, ensuring 22 ± 0.5 °C an interior temperature, and subjected to 12 h alternating periods of light and darkness. They had full access to food and drink. The experimental approach was approved in accordance with the ARRIVE guidelines (*LNCP/IAEC/23/005*).

### 4.2. Drugs

Barbaloin (purity: ≥97%) was obtained from Yucca Enterprises, India, and PTZ (purity: ≥98.0%) was procured from Sigma Aldrich, USA. All the chemicals used in this study were of excellent grade. The quantification of *IL-1β, Bcl-2, IL-6, NF-κB, caspase-3, TNF-α* and *Bax* was performed using the ELISA kit acquired from MSW Pharma, India.

### 4.3. Epileptic Seizure Scoring and a Rat Model Induced by PTZ

To induce epileptic episodes, we employed the methodology described in the study conducted by Hansen et al [61]. To initiate a dosage of 40 mg/kg, PTZ was administered intraperitoneally (i.p.) once every forty-eight hours for seventeen days until the animal displayed complete motor convulsions. Following each injection of PTZ, each rat was monitored for a period of 30 min to measure the time it took for an epileptic seizure to occur, the length of the episode, and the stage of the seizure based on the Racine scale, with some modification [51], as follows:

Level 0: No response;

Level 1: Eye and face twitching;

Level 2: Axially passing convulsive waves across the body;

Level 3: Myoclonic jerks of the body;

Level 4: Move to the side position, clonic-tonic seizures (CTS); and

Level 5: Turn over and lie on the back, GTCS, or mortality. To be completely kindled, a seizure number had to hit level 4 or 5 on three separate trials.

### 4.4. Research Design

The animals were selected randomly and then divided into four groups, with each group consisting of six animals and treated every 48 h as follows: Group I (Normal control) was administered the saline; Group II (PTZ control) PTZ was administered intraperitoneally 40 mg/kg; [62] and Group III and Group IV were administered PTZ + barbaloin 25 mg/kg and PTZ + barbaloin 50 mg/kg, respectively, for seventeen days, followed by PTZ (40 mg/kg) for another seventeen days.

At the end of the investigation, cervical dislocation was used to sacrifice the rats. The brain was excised and cleaned with a cold 0.9% NaCl solution, and then their hippocampus areas were dissected so that biochemical data could be evaluated. The tissue from the brain regions was thoroughly combined (10% weight/volume) in a buffer solution containing 0.01 M sodium phosphate (pH 7.4) and 1.15% potassium chloride per gram of tissue, ensuring a low temperature; it was then separated and, thereafter, kept at a temperature of 4 °C for enzymatic assay.

### 4.5. Behavioral Tests

#### 4.5.1. OFT

A study was conducted to ascertain the long-term impact of stress on mice. The OFT test is meant to examine the impact of PTZ on the motivational activity of animals on the final day of the trials. The unit comprises a rectangular box of 80 × 80 × 50 cm, with the floor divided into squares of equal size measuring 25 × 16 × 16 cm. The mice were placed in the central part of an open field and allowed to freely explore for a duration of 3 min. During this test, two criteria were measured, namely, the rate of locomotion, which is the number of times the mice crossed one of the four-paw grid lines. The animals were housed in the testing facility for a minimum of 2 h before the test commenced. The OFT technique was carried out in a soundproof chamber without any human involvement. Room cleaning was conducted using a 5% ethanol–water solution to eliminate any bias in behavioral testing caused by the stench left by previously used rats. A study of the motivated behavior of the mice was conducted randomly by two unbiased observers. The resulting data were statistically analyzed to assess the reliability of the interobserver test [63].

#### 4.5.2. NORT

To evaluate recognition, a novel object recognition task is used memory. In this task, rodents explore unfamiliar objects within their environment based on their innate curiosity. The purpose of this test is to determine whether a mouse is able to distinguish between familiar objects and novel objects. To start, each mouse was habituated to a 30 × 30 × 15 cm plexiglass box for 5 min. In the acquisition phase, the mice explored two identical objects for 5 min after 15 min. For mice, the objects were heavy and tall enough that they could neither move them nor climb over them. A 5-min interval was followed by mice being presented with similar objects, but with a novel or unknown object replacing one familiar object. After 5 min, the animals were allowed to explore the objects again. A discrimination index was calculated as follows: (time exploring new object − time exploring familiar object)/(time exploring new object + time exploring familiar object) [64,65].

### 4.6. Oxidative Stress Estimation

The quantification of MDA was conducted using the thiobarbituric acid technique as outlined by Ohkawa et al. in 1979 [66]. The concentration of NO in the brain supernatant was measured using the methodology established by Koracevic et al. [67]. The GSH concentration was measured using the method reported by Jollow et al. [68]. The enzymatic activity of the antioxidants CAT and SOD were determined using the methods previously described by Yousef et al. (2020) [69].

### 4.7. AChE Estimation

An approach similar to the one explained by Ellman (1961) was used to measure the level of AChE, expressed as μmol per min per mg of protein [70,71].

### 4.8. Neurotransmitter Levels

The amounts of neurotransmitters such as serotonin (5-HT), DA, and GABA were determined using high-performance liquid chromatography (HPLC).

### 4.9. Biological Inflammation

An ELISA kit was used to assess the levels of cytokines, namely, *IL-1β, IL-6, NF-κB, TNF-α, caspase-3, Bcl-2,* and *Bax.* The levels of *IL-1β, Bcl-2, TNF-α, caspase-3, Bax,* and *IL-*6 indicators were assessed in pg/mL, whereas the level of *NF-κB* was evaluated in ng/mL [72].

### 4.10. Analysis of Statistics

The data from the exams were evaluated using GraphPad software (*8.0.2*), developed by GraphPad Software Inc., California, United States. The ultimate outcomes were thereafter presented as the average value accompanied by the SEM. The results were evaluated using one-way analysis of variance (ANOVA) for the MWM test, followed by Bonferroni’s post hoc test. In addition, a one-way analysis of variance (ANOVA) was performed using Tukey’s test.

## 5. Conclusions

Barbaloin, a plant-derived natural product, has been discovered to alleviate cognitive impairments caused by PTZ. This effect is achieved by reducing oxidative stress triggered by the *NF-κβ* pathway, neuroinflammatory cytokines, and the *caspases-3, Bcl-2,* and *Bax* pathways. These findings suggest that barbaloin holds promise as a phytotherapeutic agent for treating cognitive impairments induced by PTZ in rats. Further investigation is necessary to confirm its potential neuroprotective effects in various cognitive dysfunction models.

## Data Availability

Data is contained within the article.
